# Enhancing Reliability in Redundant Homogeneous Sensor Arrays with Self-X and Multidimensional Mapping

**DOI:** 10.3390/s25133841

**Published:** 2025-06-20

**Authors:** Elena Gerken, Andreas König

**Affiliations:** Fachbereich Elektrotechnik und Informationstechnik, Lehrstuhl Kognitive Integrierte Sensorsystem (KISE), Rheinland-Pfälzische Technische Universität Kaiserslautern-Landau, 67663 Kaiserslautern, Germany; akoenig@rptu.de

**Keywords:** multidimensional mapping, Self-X system, dynamic calibration, data fusion, TMR sensors

## Abstract

Mechanical defects and sensor failures can substantially undermine the reliability of low-cost sensors, especially in applications where measurement inaccuracies or malfunctions may lead to critical outcomes, including system control disruptions, emergency scenarios, or safety hazards. To overcome these challenges, this paper presents a novel Self-X architecture with sensor redundancy, which incorporates dynamic calibration based on multidimensional mapping. By extracting reliable sensor readings from imperfect or defective sensors, the system utilizes Self-X principles to dynamically adapt and optimize performance. The approach is initially validated on synthetic data from tunnel magnetoresistance (TMR) sensors to facilitate method analysis and comparison. Additionally, a physical measurement setup capable of controlled fault injection is described, highlighting practical validation scenarios and ensuring the realism of synthesized fault conditions. The study highlights a wide range of potential TMR sensor failures that compromise long-term system reliability and demonstrates how multidimensional mapping effectively mitigates both static and dynamic errors, including offset, amplitude imbalance, phase shift, mechanical misalignments, and other issues. Initially, four individual TMR sensors exhibited mean absolute error (MAE) of 4.709°, 5.632°, 2.956°, and 1.749°, respectively. To rigorously evaluate various dimensionality reduction (DR) methods, benchmark criteria were introduced, offering insights into the relative improvements in sensor array accuracy. On average, MAE was reduced by more than 80% across sensor combinations. A clear quantitative trend was observed: for instance, the MAE decreases from 4.7°–5.6° for single sensors to 0.111° when the factor analysis method was applied to four sensors. This demonstrates the concrete benefit of sensor redundancy and DR algorithms for creating robust, fault-tolerant measurement systems.

## 1. Introduction

Driven by the progress of Industry 4.0, low-cost, compact, and portable sensors are increasingly recognized as key enablers for real-time data collection and analysis [1]. By leveraging these sensors, companies can continuously monitor equipment conditions, detect anomalies in real time to enable proactive maintenance, optimize production processes, enhance workplace safety, and substantially reduce operational costs [2]. Another advantage lies in the ability to integrate multiple sensors with minimal additional expenses, thereby facilitating more reliable measurement systems equipped with redundancy. Such a multi-sensor architecture ensures stable operation even if certain sensors fail, while also improving measurement accuracy and coverage [3,4,5,6].

However, the rapid expansion of sensor deployments introduces several challenges. First, the growing volume and complexity of data demands increased computational resources and operating expenses [7]. Second, low-cost sensors often exhibit significant limitations: excessive noise, zero drift, and susceptibility to temperature fluctuations, vibrations, and electromagnetic interference [8,9]. Such vulnerabilities can lead to inaccurate measurements, reduced reliability, and shortened device lifespans [10]. Static calibration methods, which are mostly performed only once during system initialization, are particularly ill-suited for addressing progressive sensor deterioration over time [11,12], highlighting the need for dynamic or adaptive calibration strategies.

Recent studies focusing on degradation prognosis, early fault detection, and remaining useful life (RUL) estimation [13,14,15] further emphasize the need for intelligent calibration frameworks capable of dynamically adjusting in response to predicted or detected faults.

To implement such adaptive strategies in practice, a growing body of research focuses on nature-inspired Self-X systems [16], which autonomously detect and respond to internal and external changes, emulating the adaptive mechanisms of biological organisms. Technical implementations of Self-X principles, such as self-diagnosis, self-adaptation, self-calibration, and others, have been developed to ensure reliable measurements with minimal operator intervention [17,18], which is essential in large-scale arrays of low-cost, drift-prone sensors. Building on this paradigm, researchers have introduced Self-X strategies that combine robust dynamic calibration with fault-tolerant mechanisms for real-time adaptation under evolving conditions [19,20,21,22].

A variety of machine learning (ML) approaches, such as regression models, K-nearest neighbors, random forest, support vector machines, Bayesian networks, extreme gradient boosting, generalized additive models, artificial neural networks, and deep learning, has proven effective for online dynamic calibration, with hybrid solutions further expanding these capabilities [23,24,25,26,27,28].

The development and application of these diverse ML methods reflect the growing need for robust calibration strategies capable of addressing a wide range of sensor-specific challenges. Despite methodological diversity, the overarching goal remains consistent: ensuring measurement accuracy and reliability, particularly when employing cost-effective sensors prone to degradation. Achieving this goal demands continuous, real-time adjustment of calibration parameters to effectively counteract noise, drift, and external perturbations, thereby minimizing manual interventions and operational downtime.

Among notable examples, Yang Song [29] proposes a “quasi-blind” calibration method for acoustic vector-sensor arrays, reducing the computational burden while correcting complex errors such as gain deviations and misalignment. Meanwhile, Wang et al. [30] address cross-sensitivity in infrared sensor arrays through a calibration-transfer framework, allowing a “slave” array to reuse the high-accuracy model of a “master” array via neural networks. For inertial sensor arrays, an online bias/gain correction scheme utilizes adaptive weighting based on root mean square error (RMSE) to discount degraded sensors [31]. Finally, Roos et al. [32] describe a matrix-based approach that projects multi-sensor signals onto a circular trajectory, compensating for offsets and external fields before extracting the rotation angle or linear position.

The significance of this work lies in providing a reliable and robust sensor system capable of adapting, reestablishing, and maintaining its functionality in the presence of faults or misalignments. We propose an integrated methodology for the dynamic calibration of low-cost sensors within a Self-X architecture, employing multidimensional mapping algorithms. Our experiments show that leveraging redundancy from multiple homogeneous sensors results in MAE reduction from up to 5.6° (single sensor) to as low as 0.111° (four sensors), thereby quantitatively enhancing fault tolerance.

This study builds on prior work that established a comprehensive infrastructure for Self-X sensor systems, including TMR sensor and corresponding angular decoder measurement setups [17,18,33]. As a result, the TMR angular decoder was selected as the initial research platform for benchmarking the proposed methodology, given its well-understood behavior and relevance to practical applications. Other sensor modalities will be explored in future work, and we consider the presented method transferable to a broad range of sensor systems.

A key challenge for experimental validation is that our current hardware setup includes only a single TMR sensor, while our study aims to investigate the performance of sensor arrays under various fault conditions. Analysis of the collected data shows that both amplitude and period exhibit noticeable cycle-to-cycle variations: for example, the number of samples per period ranged from 4140 to 4236 (mean 4185.2, median 4185.0), corresponding to a period variation of about 2.3%. The peak-to-peak amplitude of the differential sine signal was 2.024 V (standard deviation (SD): 0.702 V), while for the cosine signal it was 2.061 V (SD: 0.703 V). These effects complicate accurate zero-crossing detection and reliable error estimation, and prevent reliable emulation of a sensor array by combining sequential single-sensor measurements. Therefore, robust benchmarking of calibration algorithms is not feasible using such data, as too much bias would be introduced.

To address these challenges and enable a systematic and reproducible evaluation, we generate synthetic datasets that closely replicate the key characteristics of real TMR sensor signals, while eliminating undesirable time-varying artifacts. Our synthetic data closely reflect real measurements, preserving relevant error characteristics. We further validate our synthetic data using laboratory measurements and Melexis 3D simulations [34]. This dual approach enables us to emulate multiple co-mounted sensors, each with its own mechanical or electronic faults, and to benchmark calibration algorithms under controlled but realistic conditions. By carefully combining laboratory measurements and simulation results in systematic data synthesis, our approach ensures high reproducibility and comparability, crucial for benchmarking state-of-the-art calibration techniques.

The main original contributions of this study are as follows:

- A novel, flexible experimental platform enabling precise injection of controlled mechanical faults (eccentricity, air gap variations, tilt, and rotor instability) and synthetic signal distortions (amplitude imbalance, phase shifts, offsets, and noise), providing a comprehensive benchmark environment for evaluating sensor calibration algorithms.
- Realistic synthetic datasets that closely mimic real TMR sensor data, facilitating robust validation of our proposed virtual sensor array calibration framework, achieving substantial accuracy improvements (over 80% reduction in MAE) compared to a single-sensor scenario.
- A generalizable calibration methodology, demonstrated on synthetic TMR sensor data, that can be readily adapted and applied to diverse sensor types and application domains.

The remainder of this paper is structured as follows. Section 2 presents related work, including the historical development of the Self-X concept and its application in sensory electronics. Section 3 describes our proposed Self-X architecture, the experimental setup for introducing realistic TMR sensor faults, discusses common errors in TMR sensors that affect measurement accuracy, and presents the data processing workflow focusing on DR to correct systematic errors. Section 4 discusses the experimental results, demonstrating the effectiveness of the proposed solution compared to conventional methods such as static ellipse fitting. Finally, Section 5 summarizes the key findings, their practical implications, and potential directions for further research in autonomous sensor systems.

## 2. Related Work

### 2.1. Historical Development of Self-X

Over the past few decades, interest in autonomous systems capable of operating without continuous human intervention and dynamically adapting to changing external conditions has significantly increased [35,36,37]. These systems have found extensive applications in robotics, industrial automation, intelligent infrastructures, Internet of Things (IoT), cyber-physical systems (CPS), and other domains, enhancing operational efficiency, reducing maintenance costs, and enabling rapid decision-making [38].

The transition from manual processes to automation began prominently in the mid-20th century, as manufacturing facilities adopted conveyor systems, sensors, and basic control technologies [39,40]. Over subsequent decades, growing technological complexity drove the need for more sophisticated, adaptive systems capable of autonomous real-time data analysis and decision-making. Many of these approaches drew inspiration from biological systems, such as neurocomputing or organic computing, incorporating principles of self-organization and optimization [41,42,43].

A significant advancement occurred in the early 2000s with IBM’s introduction of autonomic computing, formally establishing the Self-X paradigm [44]. Autonomic computing explicitly aimed at systems capable of autonomous self-management within dynamic, unpredictable environments [45]. Concurrently, neuromorphic technology, initially designed to closely mimic neural processes through specialized hardware, faced substantial developmental challenges. Consequently, researchers shifted toward abstracting essential adaptive and parallel processing principles from biological systems to bypass the complexities of fully neuromorphic implementations [46,47,48].

Early Self-X concepts included core functionalities such as self-configuration, self-healing, self-optimization, and self-protection [49]. Over time, this scope has broadened significantly, incorporating a wide range of self-capabilities, including self-monitoring, self-tuning, self-calibration, self-maintenance, and more, continuously refining system algorithms and ensuring sustained operational accuracy [16,50,51,52]. Central to Self-X is the autonomous adaptation of internal processes to current environmental conditions and system objectives, minimizing human intervention.

From a metrological perspective, precise terminology is essential when discussing Self-X capabilities. Calibration conventionally involves systematically correcting measured values to minimize deviations without altering the measurement device itself [53]. In contrast, trimming involves physically adjusting hardware parameters to ensure accurate outputs [54]. Within Self-X, self-calibration refers to autonomously applying mathematical corrections, whereas self-trimming involves physical adjustments or reconfiguration. Advanced forms like self-repairing or self-healing represent extensive trimming methods addressing significant hardware faults [54].

Currently, Self-X research focuses on integrating predictive analytics and ML techniques to enable adaptive behavior in smart manufacturing, urban infrastructure, IoT, and CPS. The evolution of Self-X reflects both biological inspiration and pragmatic technological progression, using abstract modeling strategies to overcome hardware complexities, thus expanding automation boundaries through self-organizing and self-learning methodologies.

### 2.2. Self-X in Sensory Electronics

Sensor-based systems must consistently deliver accurate and reliable data [55]. However, low-cost sensors remain vulnerable to environmental disturbances, electromagnetic interference, and aging-related degradation [1,56]. The manual calibration and regular maintenance of these devices become economically impractical at large scales, reinforcing the need for more autonomous solutions [11,27].

Self-X methodologies specifically address these sensory challenges by offering scalable and adaptive solutions that enhance robustness, fault tolerance, and resilience. Self-X methods significantly extend sensor operational lifetimes, reduce maintenance needs, and ensure consistent reliability even under harsh conditions [57].

Fault tolerance and resilience in sensor systems typically rely on two distinct approaches:

1. Redundancy-based methods: Integrate redundant sensor modules during initial design, allowing faulty components identified by self-tests to be autonomously replaced via reconfiguration. This ensures reliability until redundancy resources are depleted but increases system complexity, size, and cost [58,59]. Unlike biological systems, technical Self-X lacks regenerative capabilities to replenish redundancy resources.
2. Soft-trimming methods: Adaptively adjust system parameters or fuse data from multiple sensors, even if they are imperfect or partially defective, using advanced adaptive fusion algorithms [32]. This paper utilizes such approaches, including DR techniques. Compensation, however, is limited by computational scheme constraints such as parameter range and resolution.

Practical implementation of Self-X methodologies in sensory electronics spans several system levels:

- Hardware level: Incorporates embedded autonomous recalibration and physical compensation capabilities directly into sensor elements and integrated circuits, mitigating inaccuracies and drift before digital signal processing [33,60].
- Software level: Employs embedded self-diagnostic and adaptive algorithms at the sensor node level, continuously monitoring sensor status, rapidly detecting anomalies, and autonomously initiating recalibration or temporary sensor deactivation [17,18,61].
- Network level: Applies self-healing and fault-tolerant techniques, activating backup sensors or employing distributed predictive modeling and interpolation to maintain uninterrupted data acquisition despite individual node failures [62,63,64].
- System level: Implements high-level self-optimization and self-adaptation strategies involving global analytics, predictive maintenance, and adaptive system-wide decision-making algorithms, ensuring overall operational efficiency and reliability [16,55].

The following sections explore advanced applications built upon these Self-X principles.

## 3. Materials and Methods

### 3.1. Proposed Self-X Architecture

**Figure 1:**
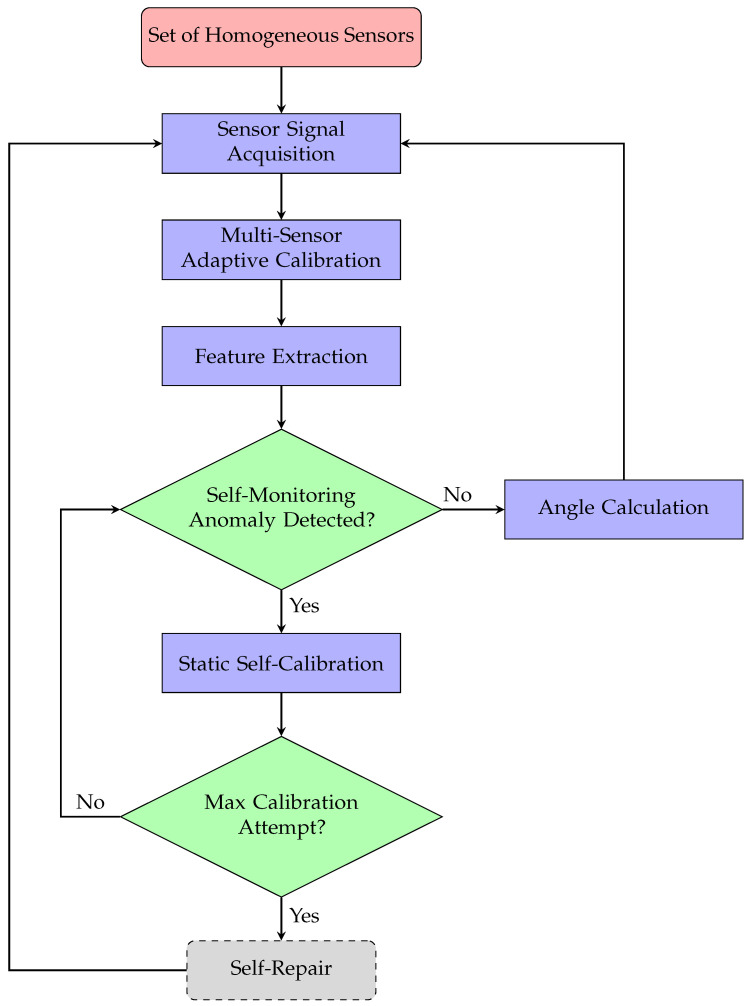
Block diagram of the proposed Self-X architecture.

The proposed methodology models a small array of homogeneous, low-cost sensors, each potentially susceptible to inaccuracies or suboptimal mounting conditions, rather than relying on a single high-accuracy sensor demanding precise and stable long-term installation. Through the application of Self-X functions, measurements from these low-cost sensors are autonomously combined to achieve robustness, accuracy, and cost-effective scalability suitable for diverse industrial environments. As illustrated in Figure 1, the output of each sensor is first processed by a reconfigurable analog front-end module, comprising an amplifier and a low-pass filter, preparing the analog signal for digitization. A field-programmable gate array (FPGA)-based Red Pitaya platform performs real-time data acquisition, employing onboard high-speed, low-noise analog-to-digital converter (ADC) to capture accurate sensor data [17].

After digitization, an adaptive self-calibration unit fuses data from multiple homogeneous sensors into a unified dataset and applies multi-dimensional mapping to project it into a lower-dimensional space. This process effectively transforms multiple sensor outputs into a single, compact representation, eliminating redundancy and enabling the system to function as a coherent virtual sensor.

The unified data next enter a feature extraction block that computes key attributes in both time and frequency domains. These attributes may include amplitude, offset, phase, spectral composition, harmonic distortion, and various statistical metrics (e.g., mean, variance, kurtosis), along with other features tailored to the specific sensors or use-case requirements. DR techniques further discard redundant or weakly informative features, minimizing the risk of overfitting and reducing computational demands, while preserving the most salient information for rapid and reliable analysis.

The extracted features are subsequently passed to a self-monitoring mechanism inspired by biological immune systems. According to immunological theory, the human immune system distinguishes between “self” and “non-self” by comparing new elements against a set of pre-learned “self” patterns. This concept underpins Artificial Immune System (AIS) algorithms, which are well suited for anomaly detection in data-limited or uncertain environments [65].

While traditional AIS approaches have primarily relied on the Negative Selection Algorithm, the methodology proposed here adopts a Positive Selection Algorithm in combination with NOVAS filtering [18]. We keep this design anchored in the established self/non-self paradigm of classical immunology, even though contemporary biological discourse increasingly frames immunity as a fluid dialogue among the host, its biome, and connectome, thereby softening the boundary between “self” and “non-self” [66]. In this framework, detectors (“antibodies”) are generated solely from normal (“self”) samples, removing the need to explicitly model fault conditions. Each new measurement (“antigen”) is compared against the trained detectors using a distance metric (e.g., Euclidean distance). If the distance to the closest detector exceeds a predefined threshold, the sample is flagged as anomalous.

Drawing on one-class classification principles, this self-monitoring approach is particularly suitable for industrial scenarios where labeled fault data are scarce, expensive to collect, or entirely unavailable [17,67].

If no anomaly is detected, the system proceeds directly to angle calculation, utilizing the freshly calibrated output of the virtual sensor. However, when an anomaly is detected, the process escalates to static self-calibration, which is performed at the software level. This calibration step, based on ellipse fitting, which is used to simulate and tune sensor outputs, improving the accuracy of angle measurements by eliminating nonlinearities and inaccuracies in sensor readings.

Should self-calibration fail to restore normal operation, the system advances to a self-repair phase. In our architecture, self-repair is conceived as a hierarchical strategy: from hardware redundancy (e.g., switching to spare sensor modules), through dynamic software reconfiguration, up to algorithmic adaptation via online parameter updates. In the present work, we explicitly demonstrate and benchmark the self-repair capability at the algorithmic level under static fault conditions, where self-repair is manifested as the system’s ability to restore or maintain measurement accuracy by adapting algorithm parameters in response to faults. Thus, our contribution extends beyond a purely conceptual framework, providing quantitative results for static scenarios.

Nevertheless, extending self-repair capabilities to dynamic, real-time fault scenarios, particularly those involving hardware-level reconfiguration, remains a key direction for future research. Such dynamic scenarios present significant challenges for realistic emulation using synthetic data or simulations alone and will likely necessitate dedicated hardware-in-the-loop experiments. By demonstrating algorithmic self-repair under controlled, static conditions, this study lays a solid foundation for future research aimed at achieving fully autonomous and robust operation in dynamic industrial environments.

### 3.2. Experimental Procedures

#### 3.2.1. Error Sources and Their Effects on TMR Sensor Accuracy

TMR sensors are widely used in automotive, aerospace, robotics, and manufacturing for applications that require precise angle measurements [68]. Even minor inaccuracies can compromise efficiency and safety in demanding environments [12]. These sensors operate as angular decoders by detecting changes in the magnetic field from a permanent magnet; the relative angle between the magnet and sensor alters the resistance, which is converted into electrical signals [69]. Two orthogonally placed sensor elements produce sine and cosine outputs, enabling angle calculation via the arctangent function. Ideally, these outputs form a perfect circle centered at (0, 0), but real-world factors introduce mechanical misalignments and circuit-level distortions that deform this circle [12,70]. Redundant configurations with multiple TMR sensors are used to increase reliability by mitigating random errors and noise [71].

**Figure 2:**
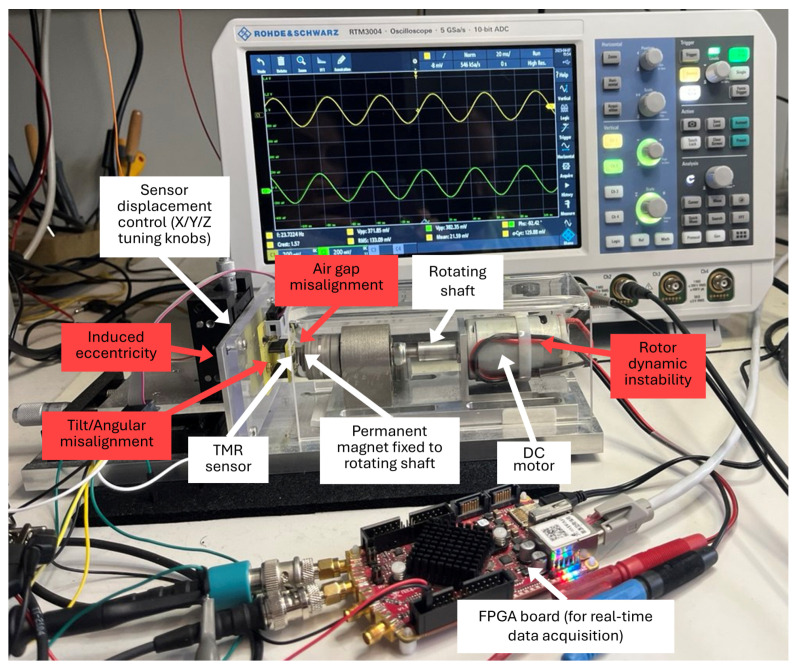
Experimental setup with fault injection capability.

The experimental setup shown in Figure 2 [33] is specifically designed to allow controlled and repeatable fault injection in TMR sensor systems. At the core of the setup is TFF953 TMR sensor (Sensitec GmbH, Mainz, Germany), powered by a 3.3 V DC supply. This sensor contains two orthogonally oriented, fully differential Wheatstone bridges, which generate analog sine and cosine outputs offset by 90° in phase and with a common-mode voltage of 1.65 V. The analog signals are digitized in real time using STEMlab 125-14 (Red Pitaya, Solkan, Slovenia) with 14-bit ADCs sampling at 15 kHz. This acquisition rate ensures accurate capture of the sensor outputs at a shaft rotation speed of 1200 rpm. More than one million samples are collected per experimental run, allowing for detailed signal analysis. Real-time monitoring and signal integrity checks are performed using RTM3004 MSO (Rohde & Schwarz GmbH & Co. KG, Munich, Germany).

Although our study employs a research prototype sensor, its main characteristics closely match those of the commercially available Sensitec TA903. According to the TA903 datasheet [72], such sensors offer a typical sensitivity of 10 mV/mT, nonlinearity error below 1.0°, and hysteresis less than 0.05°. They demonstrate high signal repeatability, as evidenced by low hysteresis and stable performance across various tests. These sensors are designed for reliable operation in a wide temperature range from −40 °C to +125 °C, and are largely insensitive to humidity due to their encapsulated design.

The sensor is mounted on a precision mechanical displacement stage equipped with micrometer tuning knobs for the X, Y, and Z axes. These knobs enable micrometer-level adjustment of the sensor’s position in all spatial directions, with a typical displacement range and step width of 10 μm. The stage makes it possible to systematically vary both the air gap and the alignment between the sensor and the permanent magnet. The sensor PCB is attached to the stage, and its tilt angle can be adjusted by varying the mounting during installation. A DC motor drives a rotating shaft to which the permanent magnet is rigidly attached. The coordinated use of the displacement stage and the rotating magnet allows the introduction of several controlled fault conditions, including:

- Induced eccentricity: Lateral displacement of the sensor or magnet relative to the axis of rotation to simulate non-concentric rotation.
- Air gap misalignment: Systematic variation in the distance (air gap) between the sensor and the permanent magnet along X, Y, or Z directions.
- Tilt/Angular misalignment: Adjustment of the sensor’s orientation to introduce tilt or angular offset with respect to the magnet.
- Rotor dynamic instability: Introduction of shaft wobble or vibrations by modifying the rotor balance or coupling.

This allows the setup to replicate a wide range of real-world faults and misalignments for robust validation of the proposed calibration approach.

To complement hardware experiments, additional signal imperfections such as amplitude imbalances, phase shifts, offsets, and added noise were simulated in post-processing. The parameter ranges for both real and synthetic disturbances were selected based on values reported in the literature [12,70,73,74].

Below, we detail the most common error types and their characteristic impact on TMR sensor output signals.

#### 3.2.2. Mechanical Failures

Mechanical failures can occur during both the installation and operation of TMR sensors. During installation, errors such as misalignment between the magnet and sensor centers, loose mounting that leads to displacement or vibration, and incorrect installation angles can introduce inaccuracies [75]. Once installed, operational factors such as housing deformation from impacts or vibrations, wear and tear altering the sensor–magnet gap, and external elements like moisture or corrosive chemicals can further disrupt sensor performance.

These mechanical issues appear as distortions in the TMR sensor’s output data, including center shifts, asymmetric deformations, and shape fluctuations. A center shift occurs when misalignment causes the sensor and magnet centers to be off-axis, hindering accurate angle measurements [75]. Mechanical deformations can create asymmetric outputs, while vibrations may scatter data points, preventing a smooth, circular shape needed for precise calculations.

**Figure 3:**
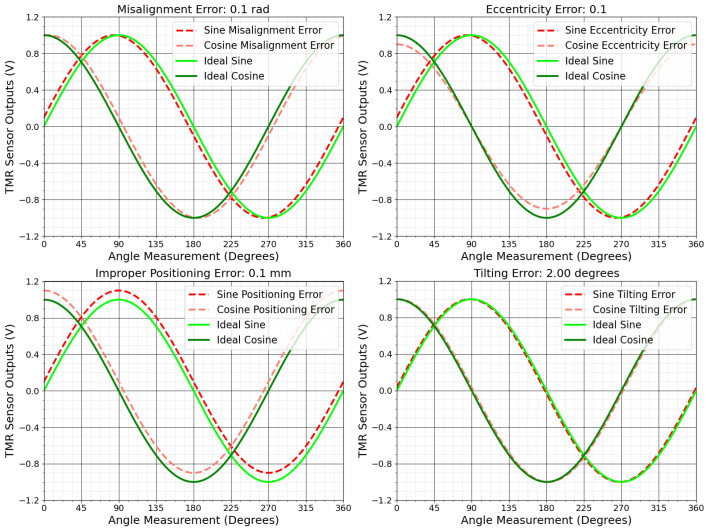
Visualization of TMR sensor output with mechanical failures.

**Figure 4:**
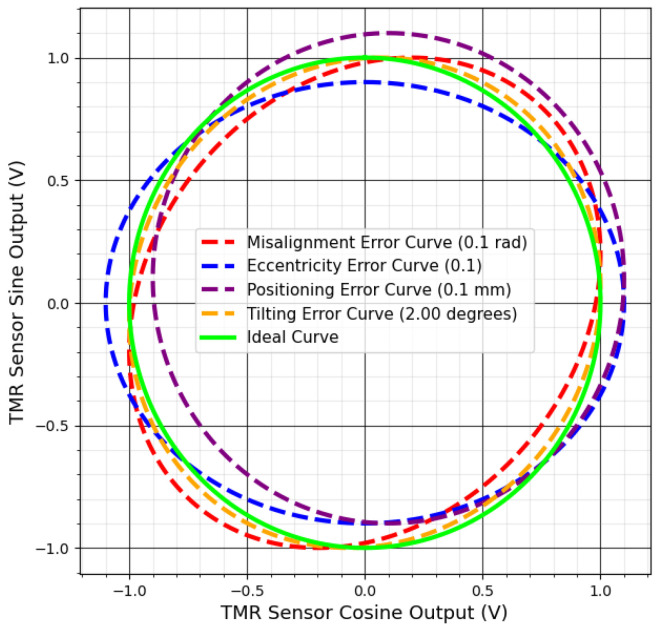
Graphical representation of distorted TMR sensor Lissajous patterns.

Figure 3 shows the effects of mechanical errors such as misalignment, positioning, and tilting on the sensor’s sine and cosine outputs. These errors, illustrated in Figure 4, distort the ideal Lissajous pattern, transforming the perfect circle into a distorted shape, which leads to inaccuracies in angular measurements.

#### 3.2.3. Measurement Failures

Measurement errors can arise from various factors during data collection and processing. Offset occurs when the sensor’s output signal consistently deviates from zero, typically due to internal sensor properties or external influences such as temperature changes, residual magnetic fields, and mechanical stresses [73]. Amplitude imbalance arises when the amplitudes of the sine and cosine signals are unequal, often caused by material heterogeneity or circuit faults [74,76]. Phase shift happens when the angle between the sine and cosine signals deviates from 90 degrees. Drift occurs over time due to changes in sensor materials or environmental conditions such as thermal expansion and material fatigue, causing gradual deviations in the output [12]. Noise, originating from electrical or environmental sources, distorts the sensor’s signals, causing fluctuations [77].

These errors appear in various ways in the output data. Drift and offset shift the center of the ideal output, creating systematic errors in angle measurements. Amplitude imbalance and phase shift alter the circle’s shape, transforming it into an ellipse, which introduces significant inaccuracies in angular calculations [74]. Noise blurs the output and scatters data points, reducing measurement precision [73].

**Figure 5:**
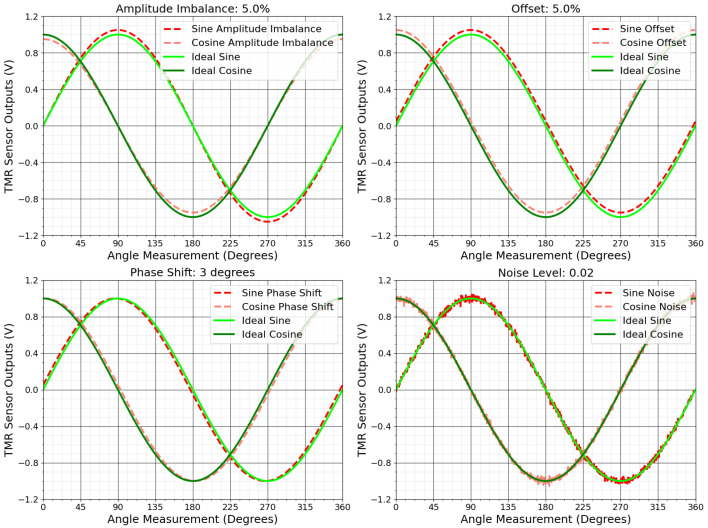
Distorted TMR sensor signals due to sources of measurement errors.

**Figure 6:**
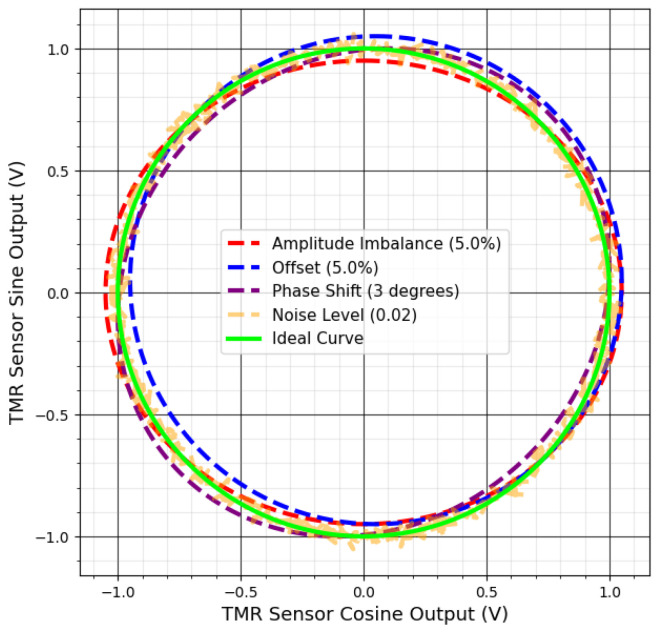
TMR sensor Lissajous curves corrupted by measurement failures.

Figure 5 shows the effects of these measurement errors on the TMR sensor’s output. Figure 6 illustrates deviations from the ideal circular shape, centered at (0, 0), for each error type.

#### 3.2.4. Circuit Failures

Circuit failures can result from issues in electronic components and connections, which critically affect measurement accuracy. Quantization inaccuracies during analog-to-digital conversion introduce uncertainties, while impedance mismatches among components may cause signal reflections and distortions. Voltage fluctuations in the power supply can alter the sensor’s output data, and component aging changes electronic characteristics over time. Shorts in tunnel junctions triggered by mechanisms similar to electrostatic discharge effectively reduce the number of functioning junctions, weakening overall signal strength [78]. Imperfect magnetization of the system magnet, sensitivity mismatches between channels, input offset, noise, signal propagation delays, and temperature changes also undermine measurement accuracy and reliability [69,76].

**Figure 7:**
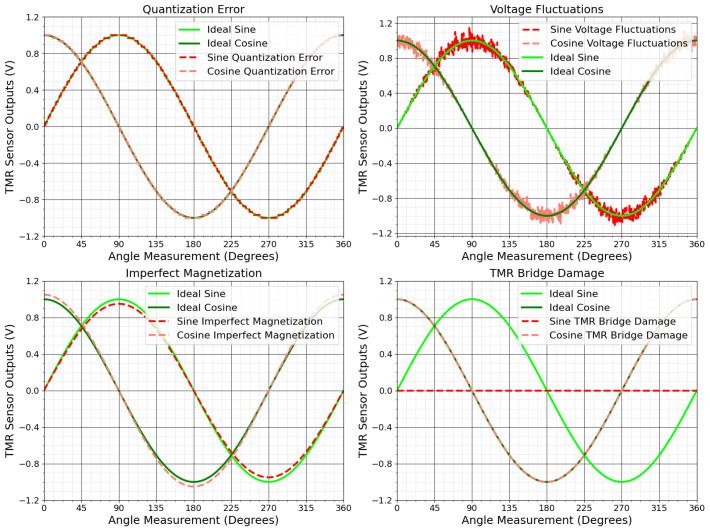
Impact of chain failures on TMR sensor outputs.

**Figure 8:**
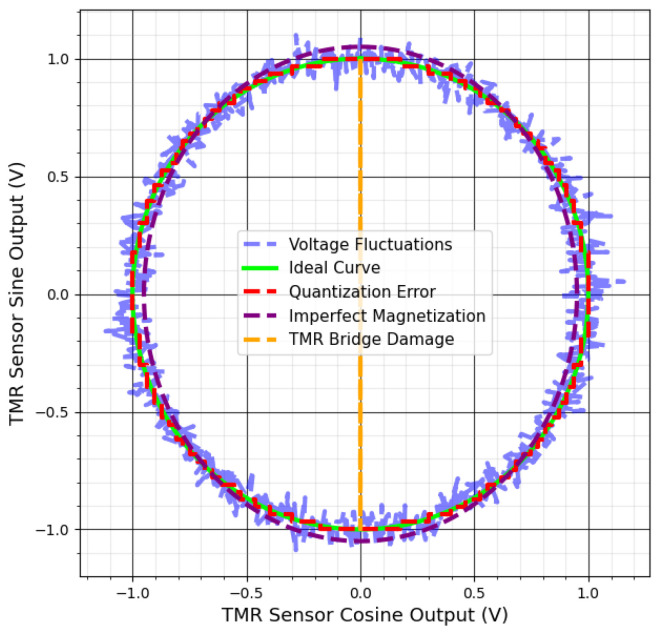
Lissajous patterns of TMR sensors with chain failures.

These circuit-related issues can produce uneven, jagged circles, prevent correct circle formation, or shift the output’s center, complicating angle determination. Figure 7 and Figure 8 illustrate how such failures scatter data points, create asymmetric circle patterns, and reduce precision. Addressing these challenges is vital for maintaining accurate TMR sensor outputs in industrial applications. The next section discusses methods to mitigate these errors and improve TMR sensor performance in practice.

### 3.3. TMR Sensor Data Processing and Dimensionality Reduction

Each TMR sensor produces sine and cosine signals over time, which can be mathematically represented as

$$p_{i}\left(t\right)=\left(p_{1i}\left(t\right),\;p_{2i}\left(t\right)\right),$$

where $p_{1i}\left(t\right)$ corresponds to the sine component and $p_{2i}\left(t\right)$ to the cosine component. For an array of *n* sensors, the measurement vector at any given time *t* is defined as

$$P\left(t\right)=\left(p_{11}\left(t\right),\;p_{12}\left(t\right),\;\ldots ,\;p_{1n}\left(t\right),\;p_{21}\left(t\right),\;p_{22}\left(t\right),\;\ldots ,\;p_{2n}\left(t\right)\right).$$

When data is collected over multiple time samples, these measurements are organized into a sensor matrix:

$$P=\left(\begin{array}{cccccccc} p_{11}\left(t_{1}\right) & p_{12}\left(t_{1}\right) & \ldots & p_{1n}\left(t_{1}\right) & p_{21}\left(t_{1}\right) & p_{22}\left(t_{1}\right) & \ldots & p_{2n}\left(t_{1}\right)\\ p_{11}\left(t_{2}\right) & p_{12}\left(t_{2}\right) & \ldots & p_{1n}\left(t_{2}\right) & p_{21}\left(t_{2}\right) & p_{22}\left(t_{2}\right) & \ldots & p_{2n}\left(t_{2}\right)\\ \vdots & \vdots & \ddots & \vdots & \vdots & \vdots & \ddots & \vdots \\ p_{11}\left(t_{m}\right) & p_{12}\left(t_{m}\right) & \ldots & p_{1n}\left(t_{m}\right) & p_{21}\left(t_{m}\right) & p_{22}\left(t_{m}\right) & \ldots & p_{2n}\left(t_{m}\right) \end{array}\right).$$

Once data is acquired, two primary approaches can be considered: data fusion (merging) and DR. Although simple fusion techniques such as averaging sensor outputs, applying a simple weighted average, or using majority voting can help reduce random noise [79,80], they still struggle to correct systematic TMR sensor errors (for example, offset drift, phase misalignment, and amplitude imbalance).

In contrast, multidimension mapping transforms the high-dimensional sensor data into a lower-dimensional space that retains the most informative features while eliminating redundancy and attenuating systematic distortions [81]. The reduced data representation significantly enhances fault tolerance, thereby allowing the system to operate reliably even if one or more sensors fail.

**Figure 9:**
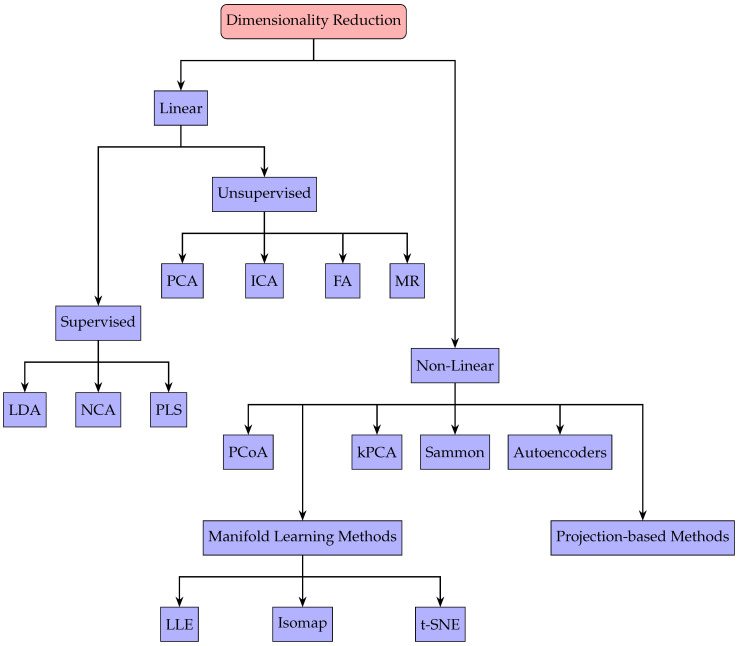
Taxonomy of linear and nonlinear DR methods, including supervised and unsupervised categories.

Figure 9 provides an overview of DR methods, divided into linear and nonlinear branches. In the linear branch, methods are further subdivided into supervised and unsupervised categories. Supervised methods rely on labeled calibration data to enhance predictive accuracy or class separation. Linear Discriminant Analysis (LDA), Neighborhood Component Analysis (NCA), and Partial Least Squares (PLS) each use labeled information to optimize how the data are projected. By contrast, unsupervised linear techniques extract lower-dimensional representations solely from patterns within the measurements. Principal Component Analysis (PCA) identifies directions of greatest variance, Factor Analysis (FA) models observed variables through fewer latent factors, Independent Component Analysis (ICA) isolates statistically independent components, and Marcus Roos’ method (MR) employs Singular Value Decomposition to perform offset and magnetic field correction [32,81].

On the nonlinear side, these methods handle more complex data relationships at the expense of higher computational requirements. Kernel Principal Component Analysis (kPCA) extends PCA through kernel functions that map data into higher-dimensional spaces, while Sammon mapping and Principal Coordinates Analysis (PCoA) each seek to preserve distance relationships in reduced form. Manifold learning algorithms like Isomap, Locally Linear Embedding (LLE), and t-distributed Stochastic Neighbor Embedding (t-SNE) uncover underlying manifold geometry by conserving local or global distances [82]. Finally, autoencoders form a projection-based family of neural network approaches that learn compressed latent representations of high-dimensional inputs without explicit labels [81,82].

As illustrated in Table 1, we provide a concise comparative overview of several DR methods [82,83]. The table compares their performance in terms of accuracy, computational efficiency, robustness, and practical implementation. This summary underscores the trade-offs between different approaches and supports our decision to employ unsupervised techniques for processing raw TMR sensor data.

In the next section, we will present the results and discussions, demonstrating how the application of these DR techniques improves the system’s fault tolerance and overall performance in angle determination under challenging conditions.

## 4. Results and Discussion

In this study, we simulated the operation of multiple TMR sensors arranged in configurations of two, three, and four units. Each sensor’s output was synthetically generated and deliberately distorted to emulate real-world error conditions (such as amplitude imbalances, phase shifts, offsets, and noise) [12,73,74]. The primary objective was to determine whether combining measurements from multiple TMR sensors provides more accurate angle estimates than using individual sensors alone.

To evaluate this hypothesis, we performed 100 simulation runs at a nominal signal amplitude of 1 V. In each run, random variations were introduced for amplitude bias (0.01 to 0.10), phase shift (0° to 2°), offset (−120 to +120 mV), and noise amplitude (0 to 5 mV) in each sensor’s output. All data in this study were generated using a simplified, stable model that excludes drift, long-term instabilities, and environmental factors. This design choice allowed us to isolate the impact of controlled electronic and mechanical distortions while eliminating the influence of more complex sensor-specific effects such as sensitivity variation, nonlinearity, and hysteresis. By focusing on this idealized scenario, we were able to clearly evaluate the core fault tolerance and robustness of the proposed approach. Future studies will expand on this work by introducing more realistic sensor behaviors and time-dependent effects.

**Figure 10:**
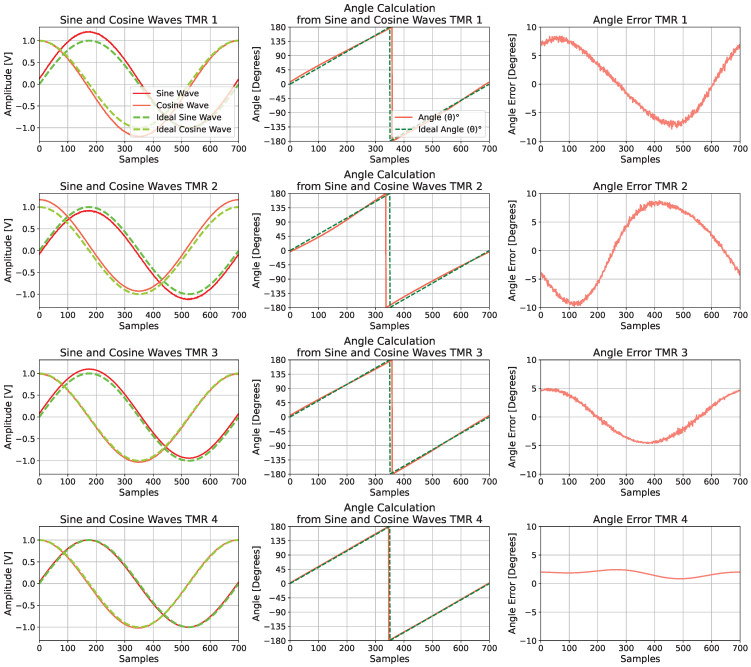
Corrupted TMR sensor outputs.

A representative combination of simulated fault parameters is illustrated in Figure 10, which is organized into three columns. The first column displays the signal amplitudes, where the sine and cosine signals for each TMR sensor are shown. The distorted signals, represented by red lines, are compared with the ideal signals, indicated by green dashed lines. The second column presents the calculated angles θ derived from the distorted signals in comparison with the ideal reference. The third column displays the resulting angular error, quantifying the deviation from the ideal case.

To assess the impact of various faults introduced into the TMR sensor signals, a set of error metrics was employed. These include MAE, RMSE, median absolute error (Median AE), maximum absolute error (MaxAE), and SD, which collectively capture both central tendency and variability in the error distribution.

Table 2 summarizes the types of faults applied to each TMR sensor, such as amplitude imbalance, phase shift, offset, and noise amplitude, as well as the corresponding angular error metrics.

**Figure 11:**
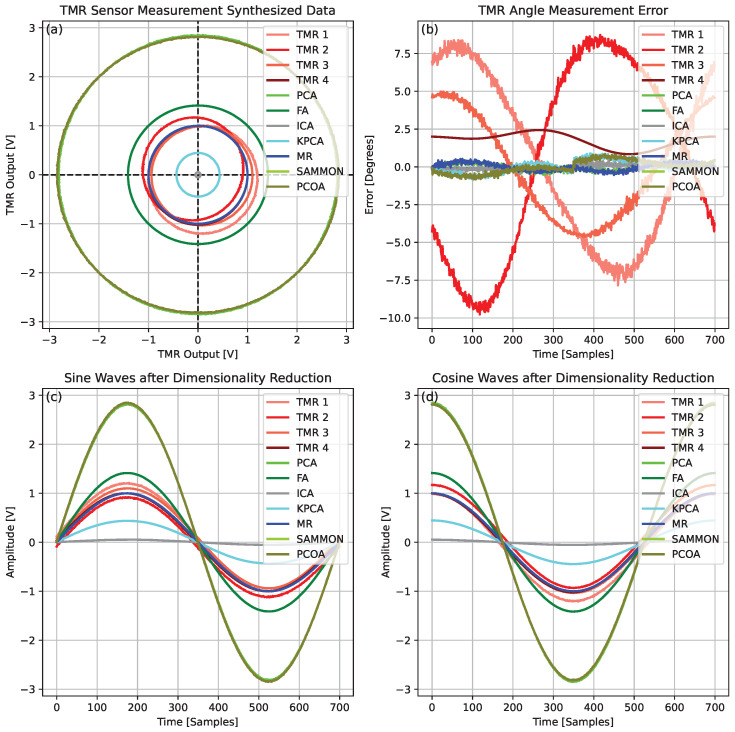
Representative signals from four TMR sensors before and after correction via DR. (**a**) Raw (distorted) and processed data, showing how each method recovers the ideal circular shape. (**b**) Angle measurement errors before and after DR, illustrating substantial improvement in accuracy. (**c**) Reconstructed sine signals, highlighting changes in amplitude and phase. (**d**) Reconstructed cosine signals, showing reduced distortion and improved alignment with the ideal reference.

The quantitative results for each DR method, obtained using the four-sensor TMR configuration, are presented in Table 3, whereas Figure 11 provides a representative visualization of the signals and errors before and after correction. FA achieved the best overall performance, producing the lowest error values. ICA, MR, and PCA also yielded competitive results with relatively low error levels. In contrast, kPCA, Sammon mapping, and PCoA showed higher residual errors, indicating reduced robustness to signal distortions.

**Figure 12:**
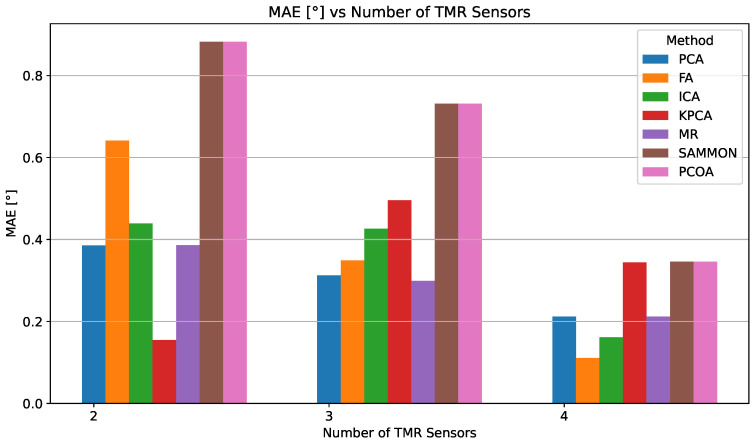
MAE comparison for different DR methods depending on the number of TMR sensors used.

**Figure 13:**
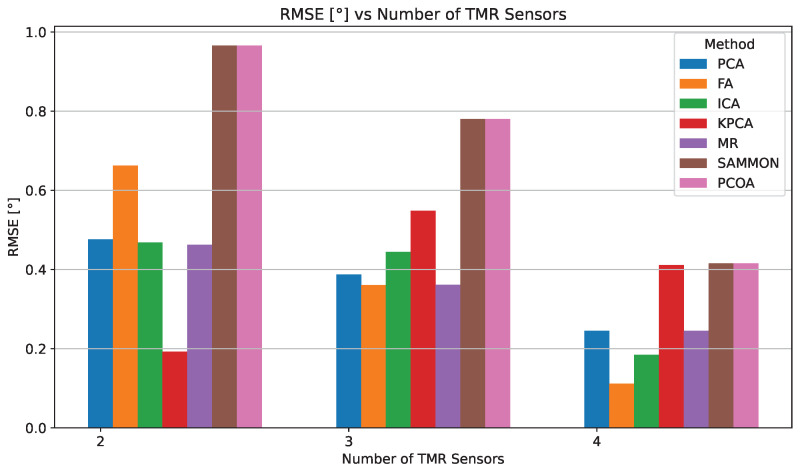
RMSE comparison for different DR methods depending on the number of TMR sensors used.

Figure 12 and Figure 13 illustrate how MAE and RMSE evolve as the number of TMR sensors increases. The detailed numerical results are provided in Table 4.

In the four-sensor configuration, FA delivers the best accuracy (0.111°), outperforming ICA (0.162°), PCA (0.212°), and MR (0.212°). Although kPCA, Sammon, and PCoA also show improvement, their MAE values remain above 0.344°, highlighting their lower resilience to signal distortions compared to linear approaches.

FA’s best performance in the four-sensor case stems from its theoretical framework, which closely matches the physical characteristics of the problem. Specifically, FA posits that a small set of unobserved latent variables can explain the correlations among observed variables [84]. In our TMR sensor array, this aligns naturally with the scenario where the eight distorted signals are noisy manifestations of two ideal, latent sine and cosine signals encoding the true angle. While PCA and similar methods seek to maximize variance, FA is designed to model the covariance structure of the data, revealing underlying factors that account for common signals and separating them from uncorrelated noise and sensor-specific errors [81]. As summarized in Table 4, the empirical results confirm this: with only two sensors, FA’s accuracy is limited (MAE 0.642°) due to insufficient information for robust covariance modeling. However, as redundancy increases to four sensors, FA effectively extracts latent variables, suppresses non-common distortions, and achieves the lowest overall RMSE (0.111°).

Beyond DR techniques, we also evaluated static ellipse fitting on the same dataset, employing the EllipseModel implementation from scikit-image [85]. As shown in Table 5, ellipse fitting markedly reduces angle estimation error relative to raw data.

Notably, in the two-sensor configuration, ellipse fitting achieves error metrics that are comparable to, or in some cases better than, several linear DR methods such as PCA, FA, ICA, and MR. However, with three or more sensors, all principal DR approaches including PCA, FA, ICA, MR, and kPCA consistently outperform ellipse fitting, achieving significantly lower MAE and RMSE values. For example, in the four-sensor scenario, the best DR method (FA) achieves an MAE of 0.111°, while the range for ellipse fitting is 0.21° to 0.72°. For completeness, polynomial regression was also tested as an alternative baseline; however, it failed to outperform either ellipse fitting or any DR method in any sensor configuration. These results indicate that while ellipse fitting is effective in reducing systematic errors, especially with a limited number of sensors, DR methods offer superior accuracy and robustness as the number of sensors increases.

This synergy between theory and experimental design accounts for the notable reduction in angular error as sensor redundancy increases. As demonstrated by our results, increasing the number of sensors and applying DR techniques reduces the MAE by approximately 11% when moving from two to three sensors, and by an additional 48% when moving from three to four sensors (see Table 2), thereby quantitatively enhancing fault tolerance and estimation accuracy. In particular, linear DR methods such as FA, ICA, MR, and PCA consistently provide robust performance in all tested configurations, underscoring their practical utility for resilient angle measurement in multisensor systems.

## 5. Conclusions

This study advances robust sensor system design by applying Self-X techniques and leveraging multiple homogeneous sensors. We investigated the benefits of fusing data from statically and, in the long term, dynamically impaired sensors by analyzing the impact of various DR methods on angular decoding accuracy in deliberately distorted TMR signals. Our results demonstrate that DR methods effectively mitigate offset, noise, amplitude imbalance, and phase shift, significantly improving data quality, measurement accuracy, and system resilience.

In summary, DR techniques provided over 80% average MAE reduction, with sensor redundancy proving key for robust angle estimation. Future work will focus on developing a real-world sensor system with integrated redundancy and dynamic Self-X capabilities to maximize reliability in the presence of sensor faults. The integration of these methods into practical applications is expected to yield valuable insights into their effectiveness and further advance the field of high-accuracy, dependable sensing.

Although this study centered on TMR sensors due to platform constraints, the proposed calibration methodology is general and can be extended to other sensor modalities. Future research will validate our approach with additional sensor types and systematically examine performance under a broader range of fault conditions, including more extreme parameter deviations, to further confirm its adaptability and robustness. 

## Figures and Tables

**Table 1:** Benchmark comparison of selected DR methods.

Benchmark	PCA	kPCA	FA	ICA	Sammon	PCoA	MR
Reconstruction Error	Low	Low	Medium	Low	Medium	Medium	Low
Computational Complexity	Low	Medium	Medium	Medium	High	High	Medium
Scalability	High	Medium	Medium	Medium	Low	Low	High
Memory Usage	Low	Medium	Medium	Medium	High	High	Medium
Robustness to Systematic Errors	High	Medium	Medium	Medium	Medium	Medium	High
Sensitivity to Outliers	Low	Medium	Medium	Low	Medium	Medium	Low
Interpretability	Medium	Low	Medium	Low	Medium	Medium	High
Implementation Complexity	Easy	Complex	Medium	Medium	Complex	Complex	Medium
Parameter Tuning	Low	Medium	Medium	Medium	High	High	Low
Real-Time Suitability	High	Medium	Medium	Medium	Low	Low	High

**Table 2:** Faults applied to each TMR sensor and corresponding error metrics.

Parameter	TMR 1		TMR 2		TMR 3		TMR 4	
	Sine	Cosine	Sine	Cosine	Sine	Cosine	Sine	Cosine
Amplitude Bias	0.1	0.1	0.015	0.05	0.02	0.01	0.0	0.01
Phase Shift [°]	1.0	0.0	1.0	0.0	0.0	0.0	2.0	1.5
Offset [mV]	100.0	−100.0	−100.0	120.0	80.0	−20.0	0.0	−10.0
Noise Amplitude [mV]	5.0	5.0	5.0	3.0	1.0	4.0	0.0	0.0
MAE [°]	4.709		5.632		2.956		1.749	
RMSE [°]	5.266		6.234		3.290		1.814	
Median AE [°]	5.101		6.274		3.250		1.890	
MaxAE [°]	8.389		9.769		4.986		2.437	
SD [°]	5.247		6.219		3.293		0.484	

**Table 3:** Comparison of angular error metrics for DR methods using four-sensor TMR configuration.

Method/Errors	MAE [°]	RMSE [°]	Median AE [°]	Max Error [°]	SD [°]
PCA	0.212	0.245	0.207	0.538	0.586
FA	0.111	0.111	0.111	0.139	0.179
ICA	0.162	0.185	0.155	0.495	0.176
kPCA	0.344	0.411	0.312	0.894	0.627
MR	0.212	0.245	0.207	0.538	0.550
Sammon	0.346	0.416	0.328	0.852	0.369
PCoA	0.346	0.416	0.328	0.852	0.369

**Table 4:** MAE and RMSE evaluation for different DR methods using two-, three-, and four-TMR sensor configurations.

Method/Configuration	2 TMR		3 TMR		4 TMR	
	MAE	RMSE	MAE	RMSE	MAE	RMSE
PCA	0.385	0.476	0.313	0.388	0.212	0.245
FA	0.642	0.662	0.349	0.361	0.111	0.111
ICA	0.439	0.468	0.426	0.444	0.162	0.185
kPCA	0.154	0.192	0.496	0.549	0.344	0.411
MR	0.386	0.463	0.299	0.362	0.212	0.245
Sammon	0.883	0.966	0.731	0.780	0.346	0.416
PCoA	0.883	0.966	0.731	0.780	0.346	0.416

**Table 5:** Angular error metrics for each TMR sensor after ellipse fitting.

Sensor	MAE [°]	RMSE [°]	Median AE [°]	Max Error [°]	STD [°]
TMR Sensor 1	0.358	0.425	0.335	1.288	0.425
TMR Sensor 2	0.715	0.810	0.745	1.633	0.810
TMR Sensor 3	0.210	0.258	0.180	0.841	0.258
TMR Sensor 4	0.241	0.268	0.268	0.388	0.268

## Data Availability

Dataset available on request from the authors.

## Abbreviations

The following abbreviations are used in this manuscript:

TMR	Tunnel Magnetoresistance
MAE	Mean Absolute Error
DR	Dimensionality Reduction
RUL	Remaining Useful Life
ML	Machine Learning
RMSE	Root Mean Square Error
SD	Standard Deviation
IoT	Internet of Things
CPS	Cyber-Physical Systems
FPGA	Field-Programmable Gate Array
ADC	Analog-to-Digital Converter
AIS	Artificial Immune System
LDA	Linear Discriminant Analysis
NCA	Neighborhood Component Analysis
PLS	Partial Least Squares
PCA	Principal Component Analysis
FA	Factor Analysis
ICA	Independent Component Analysis
MR	Marcus Roos’ Method
kPCA	Kernel Principal Component Analysis
PCoA	Principal Coordinates Analysis
LLE	Locally Linear Embedding
t-SNE	t-Distributed Stochastic Neighbor Embedding
Median AE	Median Absolute Error
MaxAE	Maximum Absolute Error
